# 17890 Cardiovascular risk factors in victims of child sexual abuse

**DOI:** 10.1017/cts.2021.743

**Published:** 2021-03-30

**Authors:** Linda R. Perez-Laras, Melissa Marzán-Rodríguez, Linda Laras

**Affiliations:** 1 University of Puerto Rico Medical Science Campus; 2 Ponce Health Science University; 3 San Juan Bautista School of Medicine

## Abstract

ABSTRACT IMPACT: The impact of this study is to encourage health professionals to screen for violent experiences as potential risk factors for CVD and adapt interventions from the non-abused in children as in adults. OBJECTIVES/GOALS: This study aims to assess the relationship between child sexual abuse and cardiovascular risk factors (CVDRF) that present in children. The objectives will provide the prevalence of CVDRF, their association with types of sexual victimization, and a score to assess the impact. METHODS/STUDY POPULATION: This study is a chart review, cross-sectional study. The Puerto Rico Health Justice Center (PRHJC) is a forensic, transdisciplinary, victim-centered, trauma-informed, and evidence-based service. The demographic variables collected are age, sex; the cardiovascular risk factors variables include a level of physical activity, tobacco exposure/alcohol, vital signs (blood pressure, BMI), lipid profile, and C-reactive protein. Sexual violence variables are the type of victimization (sexual assault, sexual molestation), the number of victimizations, and the relationship with the offender. RESULTS/ANTICIPATED RESULTS: A previous study, which examined types of evidence related to the prosecution of sexual violence cases, found that among female victims, 54% was a victim of sexual assault, and 59% had at least one health concern. The study’s hypothesis includes that older and female victims have a higher prevalence of cardiovascular disease risk factors. Also, children victims of sexual assault will have more cardiovascular risk factors than victims of sexual molestation. The age group, sex, number of victimizations, and relationship with the offender will also impact the relationship between the type of victimization and cardiovascular risk factors. DISCUSSION/SIGNIFICANCE OF FINDINGS: Early identification of child sexual abuse is needed to prevent long-term health impacts. The study’s results will be significant in developing clinical guidelines for health care providers to identify child sexual abuse as a predictor of cardiovascular risk factors and encourage victim advocates to identify cardiovascular risk factors.

